# Hemi orolingual angioedema

**DOI:** 10.4103/0972-2327.42945

**Published:** 2008

**Authors:** Dheeraj Khurana, Mukesh Sharma, Sudesh Prabhakar

**Affiliations:** Department of Neurology, Postgraduate Institute of Medical Education and Research, Chandigarh, India

A 50-year-old non hypertensive female presented with right sided hemiparesis. Her National Institute of Health Stroke Scale (NIHSS) score at admission was 12. Cranial CT was unremarkable. Her blood pressure was 200/120 mm of Hg at presentation, which was treated with IV enalapril. She was thrombolysed with IV recombinant tissue plasminogen activator (r-tPA), as per National Institute of Neurological Disorders and Stroke (NINDS) protocol. She developed hemiorolingual edema on completion of r-tPA infusion [Figure 1]. There was no airway compromise or hemodynamic instability to suggest anaphylactic reaction. Symptoms were treated with IV steroid and antihistamine. The angioedema resolved completely over the next 24 hours.

**Figure 1:**
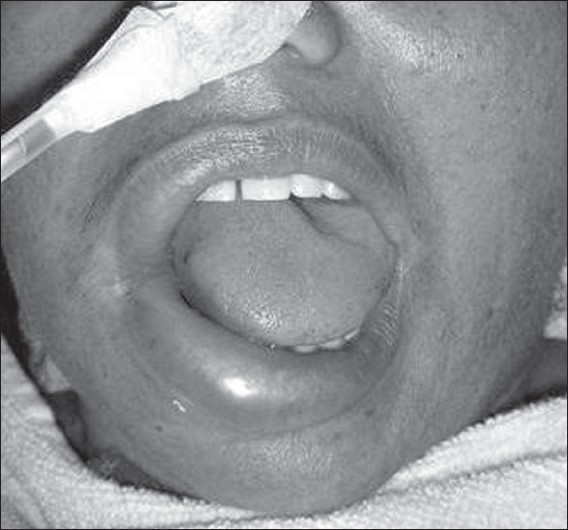
Right hemiorolingual edema following thrombolysis with IV r-tPA in patient pre-treated with IV enalapril to control hypertension

Hemiorolingual angioedema occurs in up to 5% of the patients receiving prior therapy with angiotensin-converting enzyme inhibitor (ACEi) therapy and who are administered IV r-tPA for acute ischemic stroke.[1] Angioedema may produce life threatening asphyxia, requiring intubation.[2] An increased production of bradykinin due to r-tPA, coupled with its reduced breakdown due to ACE inhibition, has been implicated in the angioedema, while an alteration in the autonomic tone, lateralized as a result of the stroke, is possibly responsible for the hemilingual onset.[1] Recognition of this uncommon condition associated with r-tPA use in acute ischemic stroke is important, as it is a reversible event, treatable with steroids and antihistamines.

## References

[1] Hill MD, Lye T, Moss H, Barber PA, Demchuk AM, Newcommon NJ (2003). Hemiorolingual angioedema and ACE inhibitor use after alteplase treatment of stroke. Neurology.

[2] Engelter ST, Fluri F, Buitrago-Téllez C, Marsch S, Steck AJ, Rüegg S (2005). Life-threatening orolingual angioedema during thrombolysis in acute ischemic stroke. J Neurol.

